# A Practical and Portable Solids-State Electronic Terahertz Imaging System

**DOI:** 10.3390/s16040579

**Published:** 2016-04-22

**Authors:** Ken Smart, Jia Du, Li Li, David Wang, Keith Leslie, Fan Ji, Xiang Dong Li, Da Zhang Zeng

**Affiliations:** 1 Commonwealth Scientific and Industrial Research Organisation (CSIRO), Lindfield, NSW 2070, Australia; Ken.Smart@csiro.au (K.S.); Li.Li@csiro.au (L.L.); d.wang@warsash.com.au (D.W.); Keith.Leslie@csiro.au (K.L.); 2 Chengdu Shuguang Optical Fiber Network Co., Ltd, 56, Tianhui Mid-street, Hi-Tech Zone, Chengdu 610041, Sichuan, China; jifan@139.com (F.J.); loricaeman@foxmail.com (X.D.L.); zengall@163.com (D.Z.Z.)

**Keywords:** terahertz, imaging, solid-state electronic components

## Abstract

A practical compact solid-state terahertz imaging system is presented. Various beam guiding architectures were explored and hardware performance assessed to improve its compactness, robustness, multi-functionality and simplicity of operation. The system performance in terms of image resolution, signal-to-noise ratio, the electronic signal modulation *versus* optical chopper, is evaluated and discussed. The system can be conveniently switched between transmission and reflection mode according to the application. A range of imaging application scenarios was explored and images of high visual quality were obtained in both transmission and reflection mode.

## 1. Introduction

Terahertz (THz) radiation, in between the millimeter-wave (100 GHz) and far infra-red (10 THz) regions of the electromagnetic spectrum, displays many unique properties, such as a strong sensitivity to polar liquids [1], high transmission through a range of non-conducting materials [2,3], and spectroscopic responses to many materials [4]. These features can be applied to medical imaging and diagnosis (for example, detecting skin cancers beneath the skin due to increased water content in tumour cells [5]), remote detection of explosive substances and drugs through spectroscopic response of crystalline compounds [3,6], and non-destructive imaging of items concealed in optically opaque packaging [2,3,7]. Recent research and achievements in THz technology are summarized in a number of review papers [4,7,8].

Since THz imaging was first demonstrated in the mid-90s [9], various technologies have been explored for this application over the past two decades. Different system architectures were built depending on what types of sources, detectors and other components were accessible. For example, typical optical-approached systems are based on various laser sources (femtosecond optical pulses, semiconductor lasers, and quantum cascade lasers) to generate and tune THz signals and photoconductive or photosensitive materials to detect/receive the signals. Time domain spectroscopic imaging and monochromatic imaging (continuous-wave (CW) imaging) [4,6,9,10] have been recognized as promising methods, especially for non-destructive inspection and materials analysis. Solid-state electronic component-based imaging systems have also been developed based on various microwave electronic components. Virginia Diodes Inc. (Charlottesville, VA, USA; http://vadiodes.com) has now offered a range of relatively mature and compact THz products such as amplifier multiplier chain THz sources and diode detectors enabling potentially compact high-performance all-electronics systems. These electronic THz imaging systems, typically in sub-THz frequency ranges, are promising for variety practical applications (for example [11]). Optic scheme is commonly used to direct and couple the THz signal to the specimen and the detector [11,12,13]. Since the solid-state electronic devices are fabricated by planar micro-lithographic processing techniques, they can be easily scaled up to large array devices for multi-pixel imaging. It is not however the purpose of this paper to provide an extensive overview of various imaging technologies, and reference [7] gives a good summary and comparison of a range of imaging technologies.

Until recent advances in THz sources and detectors, there has been a lack of low cost commercial high-performance THz instruments. Most of the THz systems presented in the literature are laboratory bench-top demonstrators; complex, bulky, difficult to operate and impossible to move. For wide adoption of THz technology to occur, the systems will have to become more compact, affordable and user friendly, which requires new approaches to many details of the components and system architectures. Some recent progress in emerging commercial THz instruments is worth mentioning. For example, Lake Shore Cryotronics, Inc. (Westerville, OH, USA; www.lakeshore.com) and QMC Instruments Ltd. (Billingshurst, UK; www.terahertz.co.uk/qmc) have offered cryogenically cooled THz spectrometers for materials characterisation, but the instruments are still bulky and expensive. Advantest Co. (Tokyo, Japan; www.advantest.com) offers a series of spectroscopic analysis systems. TeraSense Group (San Jose, CA, USA; www.terasense.com) has presented a number of THz spectroscopy systems and very recently the sub-THz imaging camera. Despite this progress, available commercial high-performance and low cost THz systems, especially imaging systems (available commercial systems are mostly spectroscopy systems), are still scarce and high cost, preventing widespread use of THz technology. Continuing research and development of THz instruments is anticipated to further drive the system costs down and promote application of the THz technology.

CSIRO has in recent years developed electronic component-based THz imaging systems [12,13], antenna-coupled integrated THz detectors [14] and sensitive superconducting detectors [15,16,17]. The previous imaging system, though producing excellent images, employed a large size backward-wave oscillator (BWO) THz source and a series of more expensive and harder to align optical mirrors to form a quasi-optical active imaging system. The system was too bulky and complicated to operate. The motivation of this work was to develop a more compact, lower cost, user-friendly and industry-adaptable imaging system. We have employed more compact state-of-the art solid-state THz components, explored various beam guiding architectures and have built a practical and simpler imaging system that could be readily applied to imaging in either transmission or reflection mode according to the desired application.

## 2. Experimental Setup—Hardware and System Configurations

Moving to a more compact system, we have first made various changes to the hardware components. The previous BWO THz source was replaced with a much more compact state-of-the art solid-state 625 GHz Amplifier/Multiplier chain (AMC) from Virginia Diodes Inc. It generates CW radiation between 590 and 650 GHz with a maximum output of around 1 mW. While the output power is less than that produced by the BWO (10 mW maximum), certain limitations of the BWO such as a finite lifespan of approximately 1000 h are overcome. The BWO has a nonstandard size output waveguide close to WR10 leading to the guide being overmoded at the test frequencies, which results in a non-ideal amplitude distribution across the collimated beam (see Figure 9a in [18]). The new solid-state AMC source does not have this overmoding issue as it has appropriately sized WR1.5 waveguide and diagonal waveguide horn with approximately 25 dB of gain. The source is also frequency sweepable and comes with a TTL modulation port. A compact economy synthesizer, instead of a standalone bench top RF signal generator, was used to supply the input signal for the AMC THz source and it offers digital tuning via LabVIEW by the system control computer. The AMC and compact synthesizer represent a great advantage over both the size, weight and complexity of the previous BWO system.

Virginia Diodes WR-1.5ZBD Zero-bias Schottky diode detectors were used to receive the transmitted or reflected signals from the sample. The detectors were mounted on a precision XY translation mounts allowing for fine adjustment of detector to optical lens alignment. The XY translator provides ±2 mm of travel perpendicular to the optical axis.

The THz imager is a quasi-optical active imaging system comprising collimating and focusing mirrors. Mirrors were used to focus the THz beam onto the sample and then onto the detector. Precision lens alignment and maintenance of these aligned lenses are important for maximizing the beam signal or signal-to-noise ratio thus obtaining the best quality images. Off-axis parabolic mirrors were used in our earlier imaging system [12,13,14,15,16]. They proved difficult and time consuming to align and realign after any movement or relocation or change of system configuration for different measurements. The off-axis parabolic mirrors were replaced with plano-convex Teflon PTFE lenses in our current new system which employed a linear arrangement of the lenses. The PTFE lenses offer the benefits of easier alignment and to a lesser extent lower cost, at only 10% of the cost of the parabolic optical mirrors. Experiments were carried out to compare both types of lenses and the beam alignment schemes. We obtained similar detectable signal amplitude and signal-to-noise ratio (SNR) from both. Although the PTFE lenses have higher loss than that of the off-axis mirrors, a simpler and better beam alignment compensated the loss. One disadvantage of the PTFE lenses over the mirrors is that visible light does not go through and therefore cannot be used for coarse alignment. This disadvantage was partially fixed in our system as described in next section by locking in all the lenses (and the detector) in the same axis, and thus removed the need for coarse alignment (only the focal distance needs fine adjustment which can be monitored with the detector signal level).

**Figure 1:**
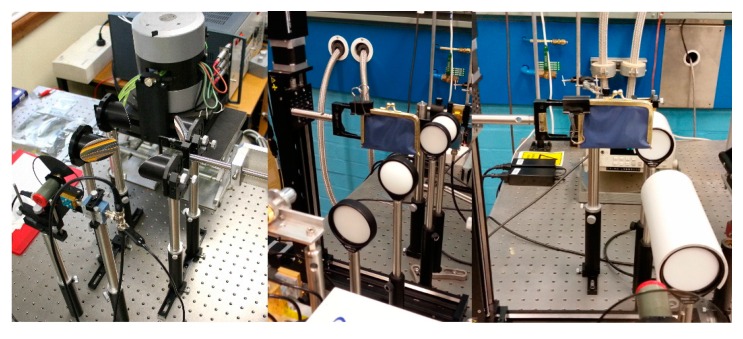
Examples of the system improvement steps with varied hardware and the beam guiding schemes.

Figure 1 shows the examples of our quasi-optical transmission imaging system improvement process. The left photograph is our earlier system using the BWO source and the off-axis parabolic mirrors. The middle picture shows a linear lens alignment scheme using the plano-convex PTFE lenses for testing the beam alignment and the image quality. The picture on the right shows the further improvement by locking in all the lenses in the same axis, *i.e.*, reducing the freedom of the movement of individual lens using a in-house fabricated PVC circular cover. Changing from the off-axis alignment to the linear system has significantly simplified the system and reduced the time required to realign the beam after the system being moved or the configuration being changed.

**Figure 2:**
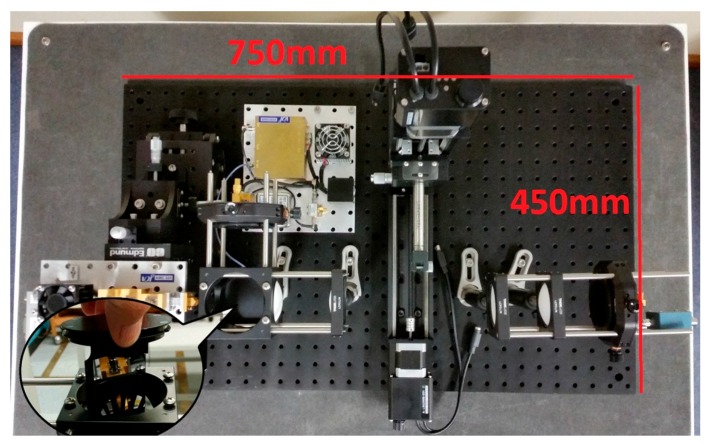
A view of the full system mounted on a portable optical plate (450 mm × 750 mm) showing a linearly aligned source-lenses-sample-detector scheme and a removable beam splitter (inset) for changing between transmission and reflection mode.

Figure 2 shows the final imaging system mounted on a portable plate without the cover. It employs a lens cage-system to lock the linear-aligned lens and is switchable between transmission and reflection imaging mode. We also designed a removable beam splitter fitted into a lens mounting cube (see Figure 2 inset) as part of the lens alignment system so the system can be easily switched from the transmission to a reflection mode. The beam splitter consists of a high resistivity float zone 0.5 mm thick silicon wafer. High resistive silicon is one of the only isotropic crystalline materials with a wide bandwidth of operation, maintaining up to 50%–54% transmission at millimetre and terahertz waves. The complete imaging system hardware is shown in Figure 2 from left to right; source, beam splitter mount (cube), reflection mode detector in a mount perpendicular to the transmission path, the frequency synthesizer above the detector, collimation and focusing lenses, the XY sample scanner, and the transmission mode detector mounted in a precision XY translation mount.

**Figure 3:**
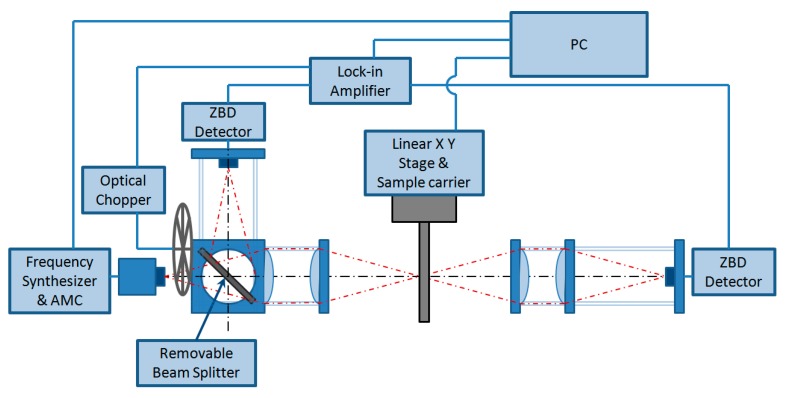
Schematic of the quasi-optical THz transmission and reflection imaging system.

The operation schematic diagram is shown in Figure 3 which illustrates how the current THz imaging system is configured. As discussed the system employs a caged lens-mounting set-up where the plano-convex PTFE lenses are mounted in a cage system formed by parallel rods. The cage mounting locks all lenses on a single axis thus reducing the freedom of individual lens movement and achieving the best lens alignment. The rigid lens mounting simplifies the alignment of the source beam to sample and then to detector and produce more robust imaging system. The system can now be moved and realigned in a matter of minutes if need be. The advantages of using such a rigid cage mounting scheme have also been recognised by Hoyer *et al.* [19] in their portable THz scanner for structural inspection of buildings.

## 3. Results and Discussion

### 3.1. Image Scanning and Resolution

An image of the measured transmission or reflection properties of the sample is generated by raster scanning the sample through the fixed focused point as illustrated in Figure 3. The THz signal generated by the AMC is collimated and focused onto the sample and then the transmitted or reflected signal is collected and focused on the detector by another pair of lenses. The sample is mounted and scanned in the X and Y planes with two moving linear translation stages. A lock-in amplifier synchronized with an optical chopper (set at 1 kHz speed) is used to acquire the detector voltage responses, which are processed by a computer to produce an image using an in-house developed LabVIEW (http://wwwni.com/labview) program.

The resolution of the system is determined by the spot size of the beam on the sample. The collimation and focusing lenses help in maximizing the beam strength from the source and minimizing the spot size on the sample. The theoretical spatial resolution can be estimated using the Rayleigh criterion ∆x = 1.22 λ*f/D* assuming the perfect optical alignment, where the wavelength λ = 3 × 10^8^ ms^−1^/614 GHz = 488 μm, *f* and *D* are the focal length and diameter of the focusing lenses. A THz signal of 614 GHz was used in the experimental results. The estimated theoretical spatial resolution is 1.19 mm. In the experiments, compromise must be made between the resolution and total scanning time. For the images shown in this paper, a typical resolution of 0.5 mm was used for a scanning area of 50 mm × 50 mm which gave an image size of 100 × 100 or 10,000 pixels. It took about 10 min to obtain the images.

**Figure 4:**
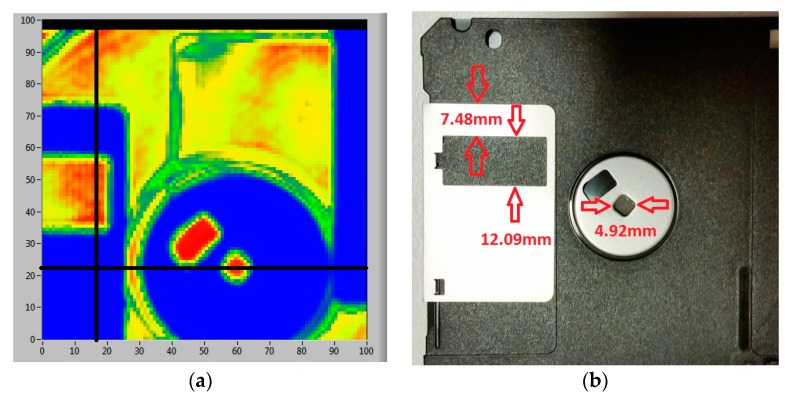
(**a**) A transmission image of a computer floppy disk; a scanned area of 50 mm × 50 mm at a resolution of 0.5 mm. The black lines indicate the single line scans at vertical line 17 and horizontal line 23; (**b**) is the photograph of the floppy disk flipped to correspond to scanned image.

**Figure 5:**
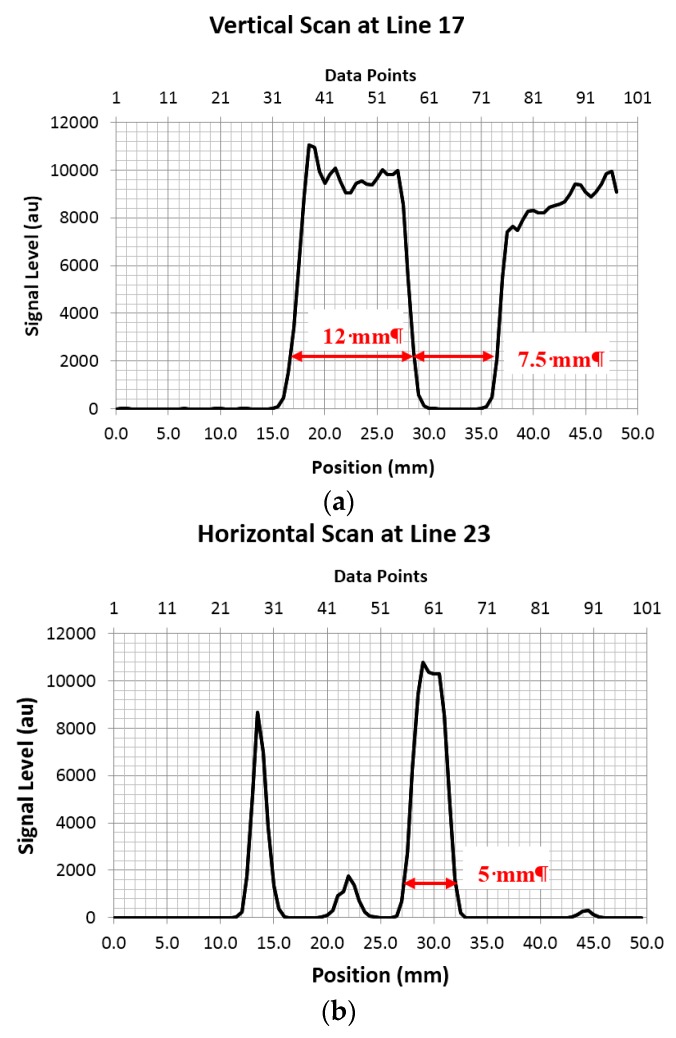
Line scans of the image data: the detected signal level of the transmitted THz beam *versus* the pixel point (upper *x*-axis) and spatial position (lower *x*-axis). (**a**) corresponds to the single line scans at vertical line 17 in Figure 4a and (**b**) corresponds to the horizontal line 23 shown in Figure 4a. The upper *x* axis shows the image pixel points (0.5 mm each step) and lower axis is converted the position points in both figures.

We examined the experimental resolution of our imaging system by plotting out 1D line scans of the image data corresponding to the image shown in Figure 4. Figure 4 is a transmission THz image of a computer floppy disk (Figure 4a) showing sharp metal edges obtained with above mentioned scanning conditions and a photograph (Figure 4b) showing the floppy disk; flipped to correspond to scanned image as the data is received on the rear of the sample. The signals blocked by the metallic parts are shown as blue colour in image (Figure 4) and correspond to zero signal level in single line scans (Figure 5). The maximum transmitted signal is shown in red colour in the image. Note that the blue section on right side was caused when the signal was blocked by a metal sample holder.

The black lines indicate the single line scans at vertical line 17 and horizontal line 23 shown in Figure 5. The imaging system scans in the vertical Y plane and steps in the horizontal *X* plane to build an image. A slight blurring at the metal edges is visible, which is caused by the scattering effect of the metal edge. This effect is minimised in our system due to the focusing effect of the convex lens; only one point of ~1 mm (theoretic prediction is 1.19 mm) in diameter is exposed to the beam and measured by the device. Therefore although the metal edges still scatter the beam this only causes slight signal dilution (green colour regions adjacent to the blue colour edges). From the image in Figure 4, the visible blurred regions (green colour) along the metal edges (vertical or horizontal edges) are approximately 2 pixels, *i.e.*, around 1 mm. The single line scans in Figure 5 correspond to the vertical line 17 across the rectangular shape metallic frame on the left side of the image and the horizontal line 23 across the small hole in the circular centre of the metallic part shown in Figure 4. The THz beam signal is completely blocked by the metallic parts resulting in zero signal level (blue in the image) and reaches the maximum signal level (red in the image) in the plastic parts of the disk which are more transparent to the THz beam. The marked widths in the line scans shown in Figure 5 show good agreement with the dimension-marked parts in Figure 4 photograph (red arrows). It can be seen that 10%–90% rise in the transmitted signal level occurs between 1.0 and 1.5 mm transition at each sharp metal edge, consistent with the visual colour change region (green) in the image (Figure 4). This provides a measure for the spatial resolution of our imaging system. The experimental results showed that our system has diffraction-limited resolution close to the theoretical spatial resolution (~1.19 mm).

### 3.2. Imaging Results in Reflection Mode

Reflection mode is suitable for many practical applications, such as security screening and non-destructive testing in industrial environments [10]. Reflection mode is particularly useful where the sample is bulky and cannot be placed at transmission focal point or where the THz radiation cannot penetrate or is largely attenuated through the sample to obtain enough signal level at the detector for quality imaging. Reflection mode is also suitable for imaging of highly reflective surfaces or for imaging the surface features or sub-surface interface of a sample; for example the detection of rust and corrosion under paint [12] and non-destructive testing of valuable historical or cultural artefacts [20].

**Figure 6:**
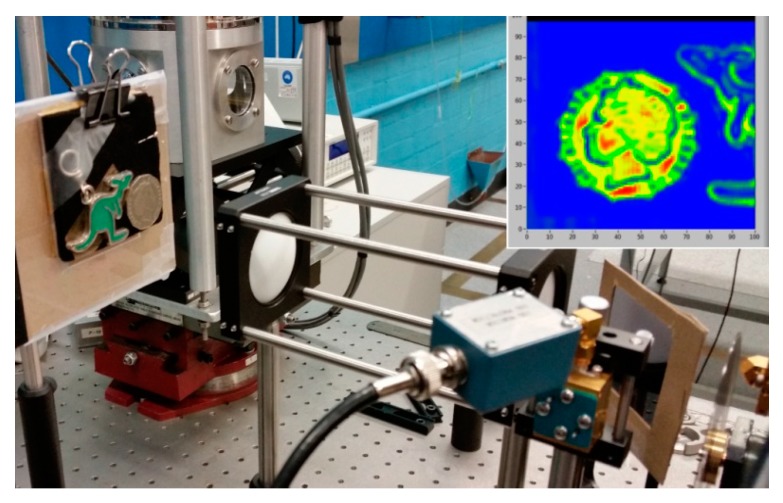
Imaging of a 50 cents coin and a Kangaroo key ring in a reflection mode (note that the experiment was carried out before the introduction of the cube mounted beam splitter shown in Figure 2).

Figure 6 shows imaging of a 50 cent coin and a kangaroo key ring in the reflection mode where a beam splitter was used to deflect the THz beam reflected from the sample surface to the detector perpendicular to the axis of the lens and original THz beam. The image obtained with the reflected THz beam is shown in the inset. It clearly shows the surface topography features of the coin and the key ring.

**Figure 7:**
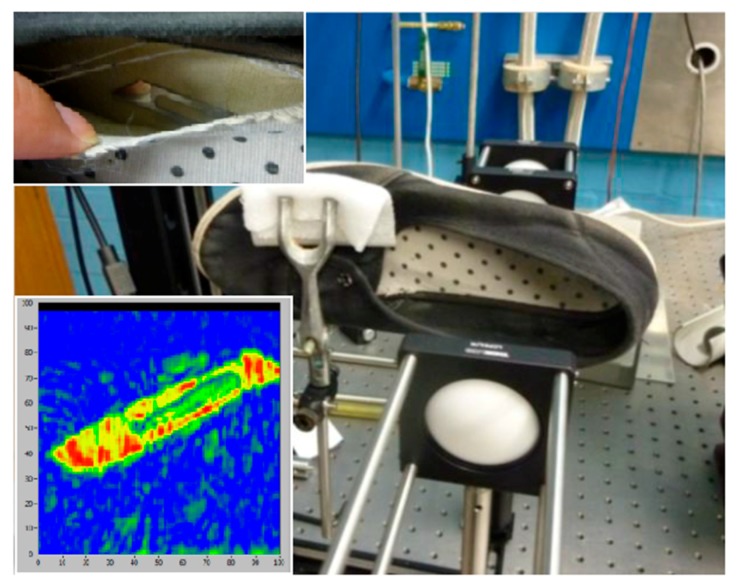
Imaging in reflection mode, showing a hidden object in shoe lining.

THz imaging has been explored for security applications such as detection of concealed weapon or dangerous objects due to its ability to penetrate through clothes and packaging materials [2,3]. Figure 7 shows another example measurement of a hidden object in a shoe using the reflection mode. The main photograph shows the setup with the shoe mounted on the XY scanner. The top inset shows a visual image of the hidden object, a scalpel inside the shoe lining material, and the lower inset shows the terahertz image of the hidden object. In the terahertz image blue corresponds to weak reflection of the THz waves and red shows strong reflection. The hidden object is clearly visible in the image demonstrating the ability of THz wave to penetrate common non-metallic materials such as the canvas cloth lining of the shoe. The result indicates the suitability of our THz imager for security applications, such as a shoe scanner in airports, for example.

### 3.3. Imaging Results in Transmission Mode

Imaging in transmission mode can be used for applications such as non-destructive testing of or inspection through opaque materials, including determining the fibre density or consistency of papers and cardboard, the detection of contaminants or inclusions in plastics and polymers. Transmission mode can also be used to quantify the water content taking advantage of terahertz frequencies’ sensitivity to water and other polar liquids. It offers a non-contact method of monitoring water content and distribution in both living things such as plants and non-living things such as wood polymers or paper [21].

**Figure 8:**
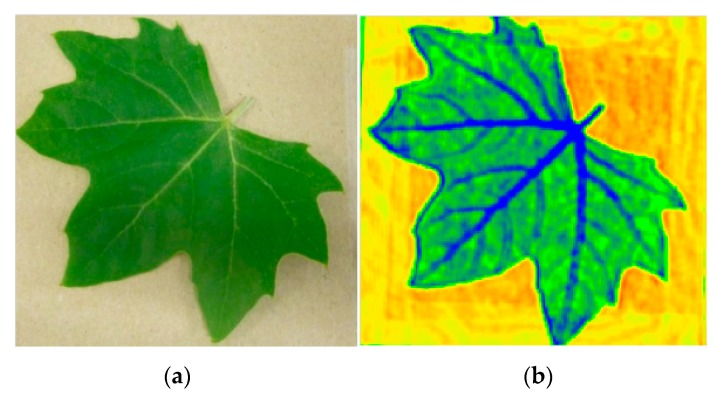
Photograph (**a**) and the transmission THz image (**b**) of a fresh leaf.

Figure 8 shows an example measurement taken in transmission mode of a fresh leaf imaged at 614 GHz; the image clearly demonstrates the key feature of the THz radiation, *i.e.* sensitivity to water. The THz image shows how the attenuation of the signal corresponds to water content and distribution particularly in the vein structure of the leaf, both large and fine vein structure can be observed. Blue colour represents the largest absorption of the THz signal and thus the highest water content. This presents a non-invasive way of monitoring water content and distribution within biological structures such as leaves. As the measurement is non-contact it can be used on live plants and repeated in situations where observing changes in water content over time are required. This example has further implication for THz imaging being applied to medical imaging examinations, with potential applications in the areas of detection of skin and breast cancers or monitoring the hydration of corneal grafts [5,22].

### 3.4. TTL Electronic Modulation versus Optical Chopper

**Figure 9:**
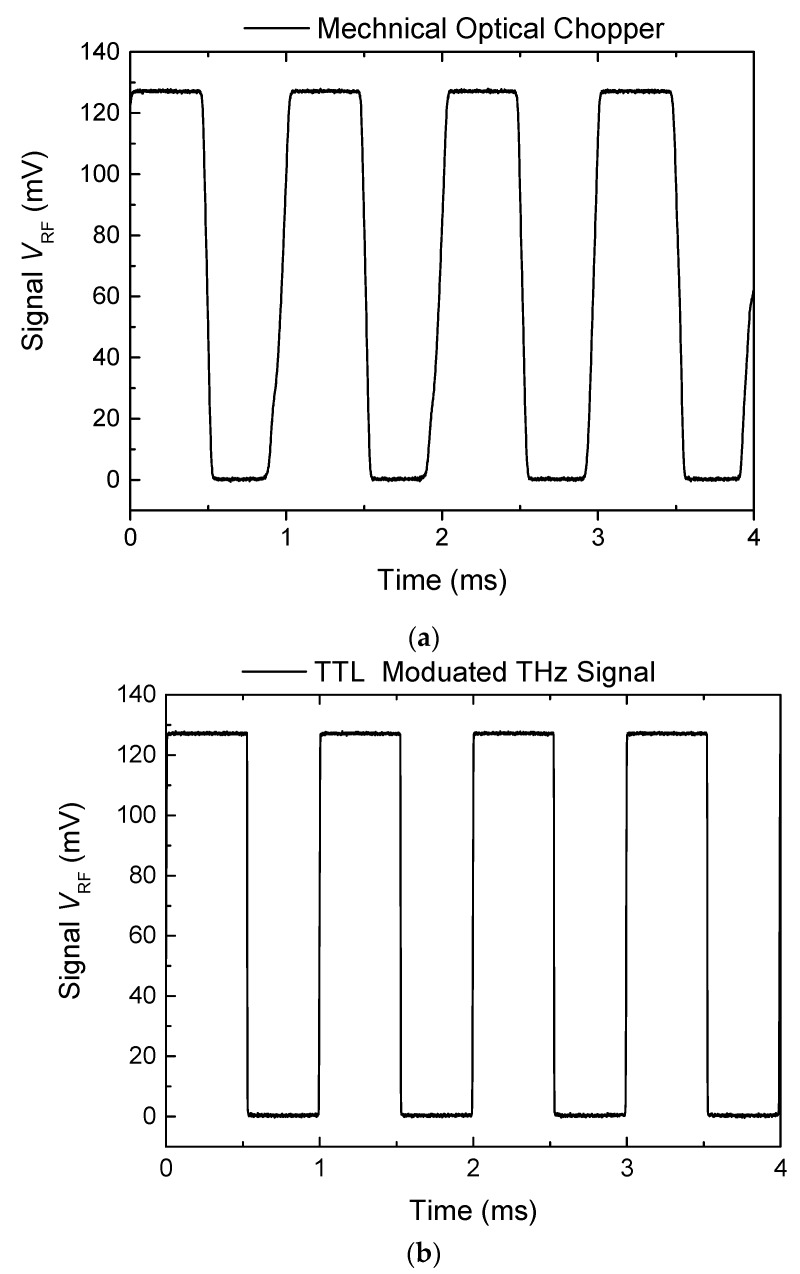
Real-time detector voltage output V_RF_ (t) traces of the modulated THz beam using a mechanical optical chopper (**a**) and an electronic signal via the TTL port on the AMC THz source (**b**).

**Figure 10:**
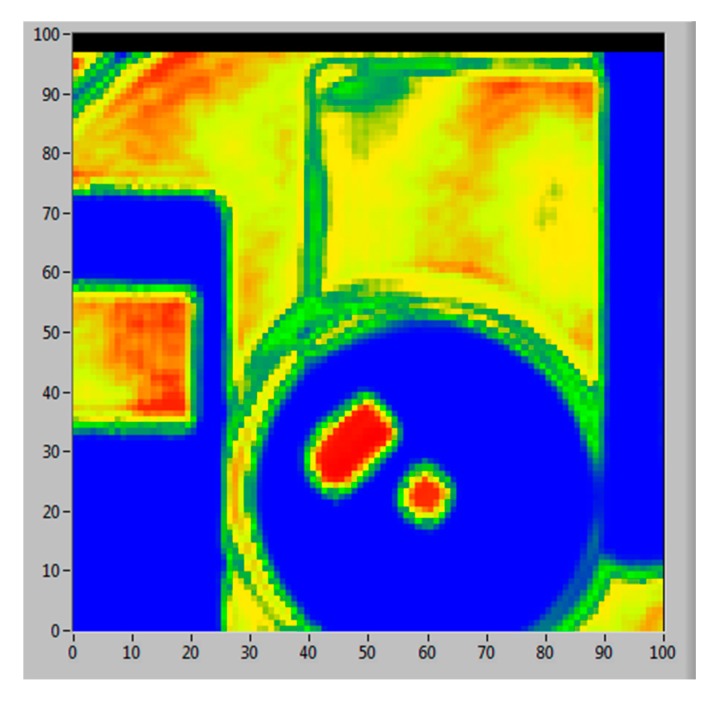
The THz transmission image of the computer disk acquired using the TTL electronic signal modulation at the same chopper speed of 1 kHz and scanning conditions as that used in Figure 4 image modulated with an optic chopper.

The above results were obtained using an optical chopper to modulate the THz source signal at the rate of 1 kHz. The ACM solid-state THz source has a logic input that allows the THz beam to be TTL modulated at up to 5 kHz. This function allows further simplification of the THz system by replacing the optical chopper with a small, low cost, logic signal source. An experiment was carried out to compare the system performance using the TTL electronic signal modulating the THz beam to that using the optical chopper modulation. Figure 9 compares the real-time detector voltage outputs (note that a different detector was used in this measurement) acquired using both modulation methods at the same rate of 1 kHz. The optical chopper modulated THz beam response shows some distortion due to the non-instantaneous nature of the optical chopping of the beam. In comparison, the TTL electronic signal modulated THz beam results in a near ideal response due to the fact that the electronic chopper can switch the RF power more quickly. The computer disk shown in Figure 4 was also imaged using the TTL electronic modulation at the same speed, 1 kHz, as that of the optical chopper, and the result is shown in Figure 10. There are no observable differences in the visual quality of these two images (Figure 4 and Figure 10), leading us to the conclusion that it is possible to replace the function of the optical chopper with TTL logic signal without compromising the system performance. Implementation of electronic modulation provides us an opportunity of developing a software-based “virtual lock-in” amplifier instead of the stand-alone analogue lock-in amplifier, which will further simplify the system and reduce the overall system cost. The development of a virtual lock-in amplifier and new signal processing method is underway.

The signal-to-noise ratio (SNR) can be estimated using the real-time voltage response V_RF_ (t) amplitude divided by the noise floor when the THz signal is blocked. The RMS noise voltage level displayed on the lock-in amplifier with the THz beam blocked is ≤0.5 μV (at integration time of 5 ms) for both electronic and choppers which are believed to be limited by the lock-in amplifier (7265 Lock-in Amplifier, Signal Recovery). The detected THz beam signal voltage output level in transmission mode is typically ~25 mV or more (with the chopper “on” but without the sample in place); giving a SNR better than 50,000 for our current system. The high SNR value results in high visual quality images.

## 4. Conclusions

A practical and portable solid-state electronic component-based THz imaging system was presented in this work. More compact state-of-art solid-state THz components were employed and various system configurations were explored to improve its performance, compactness, portability and simplicity to operate. The system can be conveniently switched between transmission and reflection mode to suit different application scenarios. The optical configuration has resulted in good spatial resolution, similar to the theoretical value of ~1.2 mm at operating frequency of 614 GHz. A SNR of better than 50,000 was obtained. Images of high visual quality were obtained in both transmission and reflection mode on a number of samples. The imaging results demonstrated some key features of THz radiation including the penetration through non-conducting material and sensitivity to water content, which indicates its potential applications in security screening, non-contact quality control and the quantification of the water content in biological tissues. The easy-to-use and portable system is a major step forward towards the industrial adoption of terahertz technology.
